# Frequency of D222G and Q223R Hemagglutinin Mutants of Pandemic (H1N1) 2009 Influenza Virus in Japan between 2009 and 2010

**DOI:** 10.1371/journal.pone.0030946

**Published:** 2012-02-17

**Authors:** Mayo Yasugi, Shota Nakamura, Tomo Daidoji, Norihito Kawashita, Ririn Ramadhany, Cheng-Song Yang, Teruo Yasunaga, Tetsuya Iida, Toshihiro Horii, Kazuyoshi Ikuta, Kazuo Takahashi, Takaaki Nakaya

**Affiliations:** 1 International Research Center for Infectious Diseases, Research Institute for Microbial Diseases (RIMD), Osaka University, Suita, Osaka, Japan; 2 Department of Virology, RIMD, Osaka University, Suita, Osaka, Japan; 3 Department of Genome Informatics, RIMD, Osaka University, Suita, Osaka, Japan; 4 Department of Infection Metagenomics, RIMD, Osaka University, Suita, Osaka, Japan; 5 Department of Environmental Pharmacometrics, Graduate School of Pharmaceutical Sciences, Osaka University, Suita, Osaka, Japan; 6 Department of Molecular Protozoology, RIMD, Osaka University, Suita, Osaka, Japan; 7 Osaka Prefectural Institute of Public Health, Higashinari-ku, Osaka, Japan; 8 Department of Infectious Diseases, Kyoto Prefectural University of Medicine, Kamigyo-ku, Kyoto, Japan; The University of Hong Kong, China

## Abstract

**Background:**

In April 2009, a novel swine-derived influenza A virus (H1N1pdm) emerged and rapidly spread around the world, including Japan. It has been suggested that the virus can bind to both 2,3- and 2,6-linked sialic acid receptors in infected mammals, in contrast to contemporary seasonal H1N1 viruses, which have a predilection for 2,6-linked sialic acid.

**Methods/Results:**

To elucidate the existence and transmissibility of α2,3 sialic acid-specific viruses in H1N1pdm, amino acid substitutions within viral hemagglutinin molecules were investigated, especially D187E, D222G, and Q223R, which are related to a shift from human to avian receptor specificity. Samples from individuals infected during the first and second waves of the outbreak in Japan were examined using a high-throughput sequencing approach. In May 2009, three specimens from mild cases showed D222G and/or Q223R substitutions in a minor subpopulation of viruses infecting these individuals. However, the substitutions almost disappeared in the samples from five mild cases in December 2010. The D187E substitution was not widespread in specimens, even in May 2009.

**Conclusions:**

These results suggest that α2,3 sialic acid-specific viruses, including G222 and R223, existed in humans as a minor population in the early phase of the pandemic, and that D222 and Q223 became more dominant through human-to-human transmission during the first and second waves of the epidemic. These results are consistent with the low substitution rates identified in seasonal H1N1 viruses in 2008.

## Introduction

In April 2009, a novel swine-derived influenza A virus (H1N1pdm) emerged and spread rapidly around the world [1], [2], causing the World Health Organization to declare a pandemic in June. To date, most countries have confirmed infections by the new virus.

Since the first appearance of H1N1pdm, one particular amino acid substitution {aspartic acid to glycine at position 222 (D222G)} within the hemagglutinin (HA) molecule has appeared sporadically [1], [3], [4], [5]. The D222G substitution is known to cause a shift from α2,6-sialic acid receptor specificity to mixed α2,3/α2,6-sialic acid receptor specificity [6], [7]. It is noteworthy that G222 is highly conserved among avian viruses [8]. Previously, α2,3-specific avian viruses have been isolated from patients during the initial phases of the pandemics of 1957 and 1968, and avian HA in humans has been shown to be selected for increased affinity for the α2,6 receptor [8]. Also, the G222 substitution was present in the Spanish Flu outbreak of 1918 [9]; however, the existence and transmissibility of H1N1pdm α2,3 sialic acid-specific viruses remain unclear.

To identify whether α2,3 sialic acid-specific viruses, which replicate well in swine, were spread during the early phase of the H1N1pdm pandemic and whether α2,3 sialic acid-specific viruses are easily transmitted, the nucleotide sequences of the HA receptor binding site of H1N1pdm in clinical specimens were determined in this study.

Several specimens obtained from mild H1N1pdm cases during the first (May 2009) and second (Dec 2010) waves of the epidemic in Japan were analyzed. In addition to the D222G substitution, we focused on two other substitutions {aspartic acid187glutamic acid (D187E) and glutamine223argine (Q223R)}, known to be critical for receptor binding, which cause a shift from α2,6 to α2,3 sialic acid receptor specificity [8], [10]. Changes in HA amino acid diversity were identified in individual cases using a high-throughput sequencer.

## Results

### Sequencing analysis of H1N1pdm HA receptor binding site (RBS) from nasal specimens in May 2009

**Figure 1:**
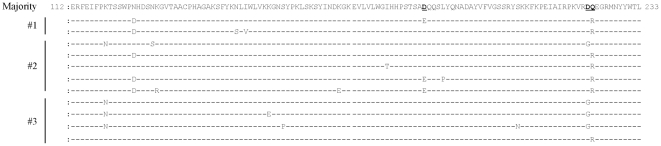
Alignment of the amino acid sequences including D187E, D222G, and Q223R mutants within the receptor binding site of H1N1pdm hemagglutinin. E187, G222, and R223 variants obtained from three clinical specimens (#1, #2, and #3) from the first wave of the outbreak. Three clinical nasal swabs were each subjected to RNA extraction, RT-PCR, and TA cloning. More than one hundred clones per specimen were sequenced using conventional Sanger technology. Positions 187, 222, and 223 are shown in bold and are underlined.

Three nasal swabs (#1–#3) from mild H1N1pdm cases were obtained in May 2009 in Osaka, Japan. Total RNA was extracted from each sample and the receptor binding site (RBS) within HA was successfully amplified using One Step RT-PCR. The PCR products (369 bp) were cloned into pGEM-T easy vector and analyzed by conventional Sanger technology. More than 100 clones in each specimen were sequenced, and the deduced amino acid sequences were analyzed (Figures 1, S1, S2, and S3). The substitution rate of the deduced amino acids was 0.29–0.41% (average: 0.34%) in the three samples, and mutations commonly observed in more than two patients are listed in Table 1. Four substitutions, K119N, N125D, D222G, and Q223R, were detected in more than 2.0% of the samples (Table 1). Among these substitutions, D222G and Q223R had already been shown to be critical for receptor binding and to cause a shift from α2,6 to α2,3 sialic acid receptor specificity [10]. In addition, the D187E substitution could be critical for the human-to-avian–type receptor switch [9]. A homology modeling and docking study also showed that D222G and Q223R mutants had reduced affinity for the α2,6 sialic acid-linked receptor (Figure S4). Thus, we focused on these five mutations and tried to measure the mutation ratio of un-isolated viruses in upper respiratory airways. For a larger scale analysis, the PCR products were examined via a high-throughput sequencing approach using a 454/Roche GS-Junior sequencer.

**Table 1:** Deduced amino acid mutations commonly observed in more than two patients in May 2009.

HA position	Wild type	Mutant	Mutant Number^a^	Ratio (%)
119	K	N	8	2.6
125	N	D	9	2.9
129	N	S	4	1.3
145	Y	H	2	0.6
147	N	D	2	0.6
154	K	E	4	1.3
157	S	P	3	1.0
160	K	E	2	0.6
179	I	T	3	1.0
182	P	Q	5	1.6
187	D	E	5	1.6
191	L	P	4	1.3
204	S	P	3	1.0
210	F	L	2	0.6
214	I	V	2	0.6
222	D	G	8	2.6
223	Q	R	10	3.2

a Number of each mutant clone out of a total of 313 sequenced clones (#1–#3 specimens).

Deep sequencing of more than 3,000 clones (reads) per specimen showed high rates of amino acid diversity, with the K119N, N125D, D222G, and Q223R substitutions in specimens #1–#3 (Table 2). The results obtained by deep sequencing were consistent with those obtained using Sanger sequencing (Table 1). The D187E substitution was also identified in two specimens through deep sequencing analysis; however, the substitution rate was only slightly increased in one of these (by 0.72% in specimen #2) (Table 2). These results suggest that α2,3 sialic acid-specific viruses, including G222 and/or R223, were present as a minor population in the upper respiratory tract even in mild cases.

**Table 2:** Frequency distribution of N119, D125, E187, G222, and R223 variants within the HA receptor binding site of viruses obtained from clinical specimens infected with influenza A viruses.

	Specimen	Total read	K119N (%)	N125D (%)	D187E (%)	D222G (%)	Q223R (%)
H1N1pdm	#1	3,308	8.59	4.99	0.15	0.74	4.64
1st wave	#2	29,607	4.61	1.01	0.72	21.49	2.39
(2009)	#3	15,514	3.45	4.88	0	3.16	3.62
H1N1pdm	#4	15,485	0.00	1.07	0	0.04	0.57
2nd wave	#5	25,429	0.00	1.33	0	0.02	0.53
(2010)	#6	14,183	0.02	2.15	0	0.07	0.47
	#7	18,046	0.01	1.87	0	0.01	0.63
	#8	11,179	3.10	0.66	0	0.05	0.48
H1N1	#9	2,014	0.00	0.00	0	0.1	0.1
Seasonal	#10	7,170	0.00	0.00	0	0.04	0.15
(2008)	#11	3,652	0.00	0.00	0	0.08	0.16
	#12	3,733	0.00	0.00	0	0.11	0.13
	#13	7,268	0.00	0.00	0	0.01	0.41
Plasmid (control)		12,428	0.00	0.00	0	0	0.38

### Multiple passages of H1N1pdm viruses in embryonated chicken eggs

To elucidate how the genomic diversity (and population) of the RBS, including the D222G and/or Q223R substitutions, might change, we inoculated these specimens into embryonated chicken eggs and serially passaged them five or six times in eggs. Hemagglutination was not detected in any samples of the first passage (P1) but was detected in the third or fourth passage and showed 2^5^ to 2^6^ hemagglutination titers on the fifth or sixth passage (P5 or P6) (Figure S5). PCR products amplifying the same region as in Figure 1 were prepared from early- and late-passaged viruses (P1 and P5/P6), as well as MDCK-passaged viruses (except #3). The amplicons, approximately 200–500 reads, were examined using deep sequencing.

D222G and/or Q223R substitutions were detected at even higher rates in the early passaged (P1) viruses (Table 3) than in the parental viruses (Table 1). More than half (98.6%, 95.0%, and 59.7% in #1, #2, and #3, respectively) possessed either D222G or Q223R substitutions (Table 3). K119N, N125D and D187E mutations were also detected at higher ratios in the P5 or P6 passages than in the P1 passage, suggesting that these mutations were being selected for effective growth in avian cells. We also detected these substitutions at a lower level in the MDCK-passaged viruses compared with the egg-passaged viruses (Table 3). In contrast to the HA gene, no amino acid substitutions were detected in the RNA polymerase B2 (PB2), RNA polymerase B1 (PB1 and PB1-F2), RNA polymerase A (PA), neuraminidase (NA), matrix proteins (M1 and M2) and non-structural proteins (NS1 and NS2) of the P5/P6 viruses compared with the nucleotide sequence of the original H1N1pdm derived from the #2 clinical specimen (data not shown).

**Table 3:** Frequency of amino acid mutations in the hemagglutinin receptor binding site in egg-passaged or MDCK-passaged samples.

Specimen	Isolation	Total Read	Mutant Ratio (%)				
			K119N	N125D	D187E	D222G	Q223R
#1	P1	495	0.6	0.2	0	3	95.6
	P6	330	0	96.7	12.1	7.9	95.8
	MDCK	481	0.2	0.8	1.5	1.9	2.7
#2	P1	340	0.6	12.1	0.3	24.4	70.6
	P6	202	60.4	33.7	25.7	60.4	33.2
	MDCK	515	0	0	0	0.4	0
#3	P1	300	0.7	0	0	59	0.7
	P5	193	49.2	36.3	0	85	0.5

P5/P6 in #1, #2, and #3 specimens reduced hemagglutination titers when RBCs were treated with α2,3 sialidase whereas the titers did not change in MDCK-passaged viruses (Table 4). These results suggested that egg-passaged viruses, including G222 and/or R223, were α2,3 sialic acid receptor tropic and that MDCK-passaged viruses including D222 and Q223 had α2,6 sialic acid receptor specificity. From these results, we can extrapolate that the major populations in the original specimens #1–#3 had α2,6 sialic acid receptor tropism. These results are coincident with the homology modeling and docking of D222G and Q223R mutants (Figure S4).

**Table 4:** Hemagglutination assay using chicken, guinea pig, and horse erythrocytes with or without α2,3 sialidase treatment.

	Chicken		Guinea pig		Horse	
α2,3 sialidase	−	+	−	+	−	+
#1-P6	512	8	1,024	32	128	16
#2-P6	512	256	512	1,024	64	4>
#3-P5	256	128	512	512	64	4>
#1-MDCK	256	256	512	1,024	128	128
#2-MDCK	32	16	256	256	8	8

### Deep sequencing analysis of H1N1pdm HA D222G or Q223R mutants from the second wave of the outbreak

To elucidate the transmissibility of the G222 and/or R223 viruses in humans, five nasal swabs (#4–#8) obtained from individuals with a mild case of H1N1pdm in December 2010 were examined by deep sequencing analysis. The same region of HA RBS (369 bp) as shown in Figure 1 was amplified by One Step RT-PCR. The results showed that, by December 2010, both the G222 and R223 variants had almost disappeared (0.01–0.07% and 0.47–0.63%, respectively) (Table 2). The K119N mutation frequency also shifted to an undetectable level except for one specimen (#8). By contrast, the N125D mutation could still be detected (0.66–2.15%; average, 1.42%) (Table 2). Taken together, these results suggest that the H1N1pdm D222 and Q223 variants are competent for human-to-human transmission but that the α2,3 sialic acid-specific G222 and R223 mutations resulted in low transmissibility. We also examined the substitution rates in seasonal H1N1 viruses using five nasal swabs (#9–#13) from patients diagnosed in 2008 (Table 2). The results showed low substitution rates for D222G and Q223R (0.01–0.11% and 0.1–0.41%, respectively), consistent with the results of the second epidemic, in H1N1pdm.

## Discussion

This study used a high-throughput sequencing approach to analyze more than 2,000 clones from each of eight H1N1pdm samples obtained in 2009–2010. The results showed that α2,3 sialic acid-specific viruses containing D222G and/or Q223R substitutions within the HA molecule were present in the upper respiratory tract as a minor population in patients with mild H1N1pdm infections in the early phase (May 2009) of the pandemic in Japan. However, these substitutions nearly disappeared in the samples from five mild cases in Dec 2010, suggesting that the D222G and/or Q223R mutants showed low rates of human-to-human transmission. Thus, the newly emergent influenza A viruses may have been dual specific but not exclusively α2,6-sialic acid-specific during the early phase of the pandemic and adapted during multiple cycles of human-to-human transmission. These results are consistent with previous reports showing that, during previous pandemics, the receptor binding properties of avian influenza viruses changed after introduction into mammals [8]. In particular, positions 187 and 222 within the HA of H1 viruses and position 223 in H2 and H3 strains are critical for receptor binding specificity [8]. In the present study, the D187E substitution was not striking, suggesting that E187 had already disappeared in humans, or the original swine lineages, by May 2009. The N125D mutation, however, was detected (0.66–2.15%), even in the second wave of the outbreak (Table 2). However, as far as we know, there is no report indicating that this mutation affects receptor-binding affinity and specificity. The K119N mutation was also significantly detected in one specimen (3.1%) from a patient who had similar clinical symptoms as those of other patients during the second wave of the outbreak (Table 2). Neither mutation was detected in specimens of seasonal H1N1 in 2008, suggesting that further prospective study, as well as virological study, is required to understand the functions of these mutations.

HA residue 222 plays a critical role in the binding affinity of the galactose moiety of sialosaccharides for the RBS. The D222G mutation would result in a loss of interaction between galactose and K219 (Figure S4) because of the loss of charge and the side-chain. In turn, this would open up a cavity on the side of the RBS to accommodate the α2,3-linked receptor [11], [12]. Residue 223 is also important for the binding affinity of galactose and the terminal sialic acid of the receptor. The emergence of a positively charged and bulky Arginine(R) instead of Glutamine(Q) at this position would disturb the interaction between K219 and galactose, leading to decreased virus affinity for the α2,6-linked receptor (Figure S4) [13], [14]. Also, the homology modeling results showed that the double mutations, D187E and Q223R, would decrease virus affinity for the α2,6-linked receptor because the salt-bridge between E187 and R223 would lead to narrowing of the receptor binding pocket (data not shown).

In this study, we performed deep amplicon sequencing using Roche/454 GS-Junior technology. We compared the results ob<1?show=[dh][dh]?>tained by using deep sequencer with those obtained by the conventional Sanger method to evaluate this newly developed technology. Sequencing analysis using next-generation technology is necessary to reveal H1N1pdm genetic diversity in detail; our preliminary sequencing analysis using a conventional cloning approach failed to detect the D222G mutation in specimen #1 (Figure 1 and Table 2). The frequency of mutations such as D187E in specimens #1 and #2 and D222G in specimen #2 was not the same between GS-Junior technology and conventional Sanger sequencing (Figures 1, S1, S2, and S3, Tables 1 and 2). This discrepancy might be due to the different number of clones sequenced in each method. The proportion of HA sequences observed with D222G and Q223R in the #1 and #2 P6 and in the #3 P5 PCR products (Table 3) was consistent with results obtained by direct sequencing analysis using the ABI Sanger sequencer (Figure S6), suggesting that high-throughput amplicon sequencing analysis was highly quantitative.

The present study did not confirm whether viruses with the D222G and/or Q223R substitutions actually replicated in humans; however, the D222G and Q223R mutant viruses formed the dominant population when specimens were inoculated into embryonic chicken eggs (Table 3), indicating that the G222 and R223 mutants may be able to replicate in humans.

Several reports have shown that the D222G substitution was associated with severe, and sometimes fatal, cases of H1N1pdm [10], [15], [16], [17], [18]. It has also been reported that two different amino acids, D and E, may be polymorphic variants at position 222 [19], and that amino acids, G or N, would be present in potentially more pathogenic mutants or circulating variants [20]. However, the relevance of the mutation at position 222 to H1N1pdm pathogenesis remains unclear. In this study, D222E, D222N, and D222V variants were not detected, even when deep sequencing was performed (Table S1). D222G mutants were detected as a minor population even in mild cases; thus, every patient may have been exposed to D222G mutant viruses. Minority α2,3 sialic acid-tropic G222 mutants could be amplified in α2,3 sialic acid expressing cells in the lower respiratory tract of certain populations, which would result in severe to fatal respiratory illness in these populations. No conclusions can be drawn regarding the relationship between the D222G substitution and pathogenesis from the present study. Therefore, further study is required to determine whether G222 viruses were ever present as a major population in the lower respiratory tract of patients with severe or fatal pneumonia due to H1N1pdm infection.

## Materials and Methods

### Clinical samples

Three and five individual H1N1pdm-positive nasal swabs were provided in May 2009 and December 2010, respectively, in Osaka, Japan. All of the patients examined in 2009 had fever, sore throat, and cough, but were not hospitalized. They were administered oseltamivir after nasal swab-sampling. The patients examined in 2010 had upper respiratory symptoms but were not hospitalized. As a control, five specimens diagnosed with seasonal H1N1 in 2008 in Osaka were investigated. The analyzed samples were unlinked and anonymous in the Osaka Prefectural Institute of Public Health. This study was approved by the ethical review committees of the Osaka Prefectural Institute of Public Health and the Research Institute for Microbial Diseases (RIMD), Osaka University. The Both ethical committees specifically waived the need for consent.

### RNA extraction

Each nasal swab was collected in Hanks solution and was centrifuged at 20,000 *g* for 10 min. The supernatants were suspended in TRIzol LS reagent (Life Technologies/Invitrogen, Carlsbad, USA) for 60 min. Total RNA was extracted using a PureLink™ RNA Mini Kit (Life Technologies/Invitrogen) according to the manufacturer's instructions. Contaminating DNA was eliminated with DNAase I (Life Technologies/Invitrogen).

### One-step reverse transcription-PCR (RT-PCR)

Total RNA was subjected to One-step RT-PCR (SuperScript III/Platinum Taq HiFi One-step RT-PCR Kit; (Life Technologies/Invitrogen) to detect the receptor binding site in the HA molecule. The primers used were as follows: forward primer, 5′-_416_ TTGAAAGGTTTGAGATATTC_435_-3′; reverse primer, 5′-_784_ CTAGTGTCCAGTAATAGTTC_765_-3′.

### Plasmid cloning

Amplified PCR products were cloned into the pGEM-T easy vector (Promega, Madison, WI). More than one hundred clones per specimen (109, 103, and 101 clones in #1, #2, and #3, respectively) were sequenced using conventional Sanger technology (Life technologies/Applied Biosystems, Carlsbad, USA).

### Virus isolation and serial passage in embryonated eggs and Madin-Darby canine kidney (MDCK) cells

Clinical specimens were injected into 9-day-old embryonated chicken eggs for virus isolation [21]. After incubation at 37°C for 72 h, the allantoic fluids were collected and filtered (Passage 0: P0). The samples were then diluted 1–100 fold in phosphate buffered saline (PBS) and passaged either six (samples derived from #1 and #2) or five (sample derived from #3) times in embryonated chicken eggs. Identical specimens derived from #1 and #2 were also inoculated into MDCK cells for virus isolation [22] and the amplified viruses were serially passaged two or three times in MDCK cells.

### Hemagglutination titration

Viral samples were serially diluted with PBS and added at a concentration of 0.5% to chicken red blood cells. After incubation at room temperature for 30 min, hemagglutination was observed.

### High-throughput pyrosequencing and data analysis

PCR products were purified using the QIAquick® PCR purification kit (Qiagen GmbH, Hilden, Germany) and were ligated with RL MID adaptors (Roche Diagnostics GmbH, Mannheim, Germany). The PCR products were further purified with Agencourt AMPure XP beads (Beckman Coulter, Brea, USA). The quality and quantity of the library was assessed using an Agilent 2100 Bioanalyzer (Agilent Technologies, Santa Clara, USA) and TBS380 mini fluorometer (A. Daigger & Company Inc., Vernon Hills, USA), respectively. After amplifying the samples by emulsion PCR, high-throughput pyrosequencing was performed using a GS Junior (Roche Diagnostics GmbH). Data analysis was performed on each sequence read using computational tools as described previously [23], [24], [25].

### α2,3 sialidase treatment

100 µl of 10% chicken, guinea pig, or horse red blood cells were treated with 10 U of α2,3 sialidase (New England Biolabs, Ipswich, MA) at 37°C for 18 h and were washed with PBS. After the erythrocyte suspension was diluted to 0.5% (chicken) or 0.75% (guinea pig and horse), the hemagglutination titer was measured as described above.

### Homology modeling and docking

The crystal structure of HA of influenza virus, A/Brevig Mission/1/1918 (H1N1) (Protein Data Bank ID code 2WRG), was used as a template for homology modeling by using a Molecular Operating Environment. SA α2,6-linked structural analogs were used as inputs for docking with the model HA structure.

## Supporting Information

Figure S1 **Alignment of the amino acid sequences within the receptor binding site of variants other than E187, G222, and R223.** Variants other than E187, G222, and R223 (Figure 1) obtained from clinical specimen #1 from the first wave of the outbreak.
(TIFF)

Figure S2 **Alignment of the amino acid sequences within the receptor binding site of variants other than E187, G222, and R223.** Variants other than E187, G222, and R223 (Figure 1) obtained from clinical specimen #2 from the first wave of the outbreak.
(TIFF)

Figure S3 **Alignment of the amino acid sequences within the receptor binding site of variants other than E187, G222, and R223.** Variants other than E187, G222, and R223 (Figure 1) obtained from clinical specimen #3 from the first wave of the outbreak.
(TIFF)

Figure S4 **Stereo views of the homology-based structural model for binding of sialosaccharides to the RBS of H1N1pdm hemagglutinin.** The structural complex of the RBS of H1N1pdm HA with human receptor (orange) was constructed using the co-crystal structure of A/Brevig Mission/1/1918 (PDB ID: 2WRG) as a template. (A) HA of wild type (pink) overlapped with the HA of D222G mutants (green). Position 222 is shown in deep colors. Putative interactions salt-bridge between the sugar and the RBS are shown as black dashed lines. (B) HA of wild type (pink) overlapped with the HA of Q223R mutants (blue). Q223 and R223 are shown in deep pink and yellow, respectively.
(TIFF)

Figure S5 **Kinetics of hemagglutination in viruses isolated from egg-passaged samples (#1, #2, and #3).** Clinical specimens were injected into 9-day-old embryonated chicken eggs and incubated for 72 h. Allantoic fluid was collected (passage 0) and serially passaged in chicken eggs (passage 5–6). The viral growth of each sample was measured by hemagglutination assay using (0.5%) chicken red blood cells.
(TIFF)

Figure S6 **Direct sequencing analysis of PCR products (positions 221–227 aa in HA-RBS) amplified from egg-passaged P6 (#1 and #2) and P5 (#3) viruses using a conventional ABI sequencer.** The arrows indicate nucleotide substitutions. WT indicates wild type.
(TIFF)

Table S1 Frequency distribution of D222G, D222N, D222V, and D222E variants within the hemagglutinin receptor binding site of viruses obtained from clinical specimens (#1–#3).
(TIFF)
