# Supplementary figures and images for: Frequency of D222G and Q223R Hemagglutinin Mutants of Pandemic (H1N1) 2009 Influenza Virus in Japan between 2009 and 2010
Source: PLoS One. 2012 Feb 17;7(2):e30946. doi: 10.1371/journal.pone.0030946 (PMC3281909; doi:10.1371/journal.pone.0030946)

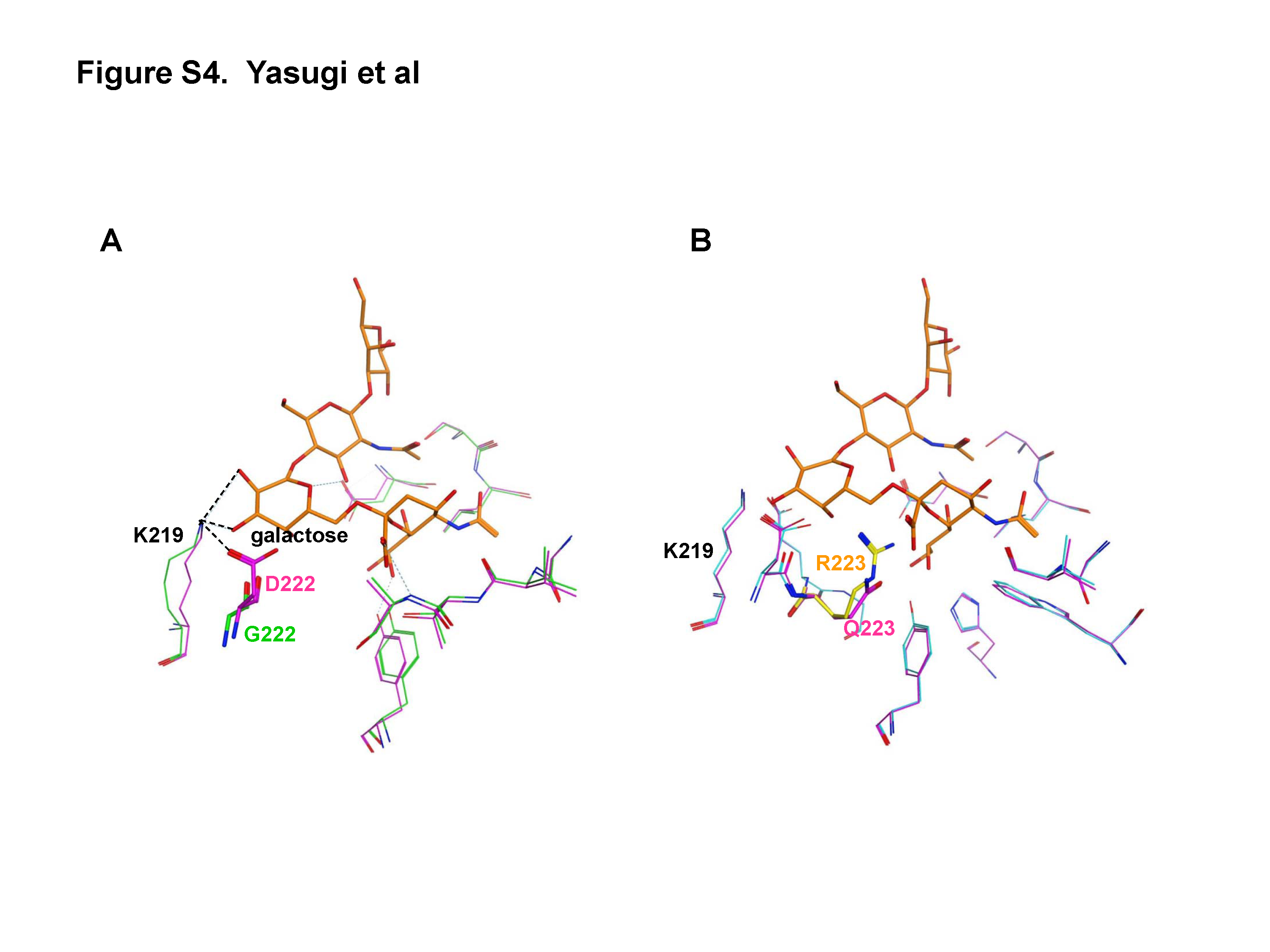

Supplement: Figure S4 — Stereo views of the homology-based structural model for binding of sialosaccharides to the RBS of H1N1pdm hemagglutinin. The structural complex of the RBS of H1N1pdm HA with human receptor (orange) was constructed using the co-crystal structure of A/Brevig Mission/1/1918 (PDB ID: 2WRG) as a template. (A) HA of wild type (pink) overlapped with the HA of D222G mutants (green). Position 222 is shown in deep colors. Putative interactions salt-bridge between the sugar and the RBS are shown as black dashed lines. (B) HA of wild type (pink) overlapped with the HA of Q223R mutants (blue). Q223 and R223 are shown in deep pink and yellow, respectively. (TIFF) [file pone.0030946.s004.tiff]

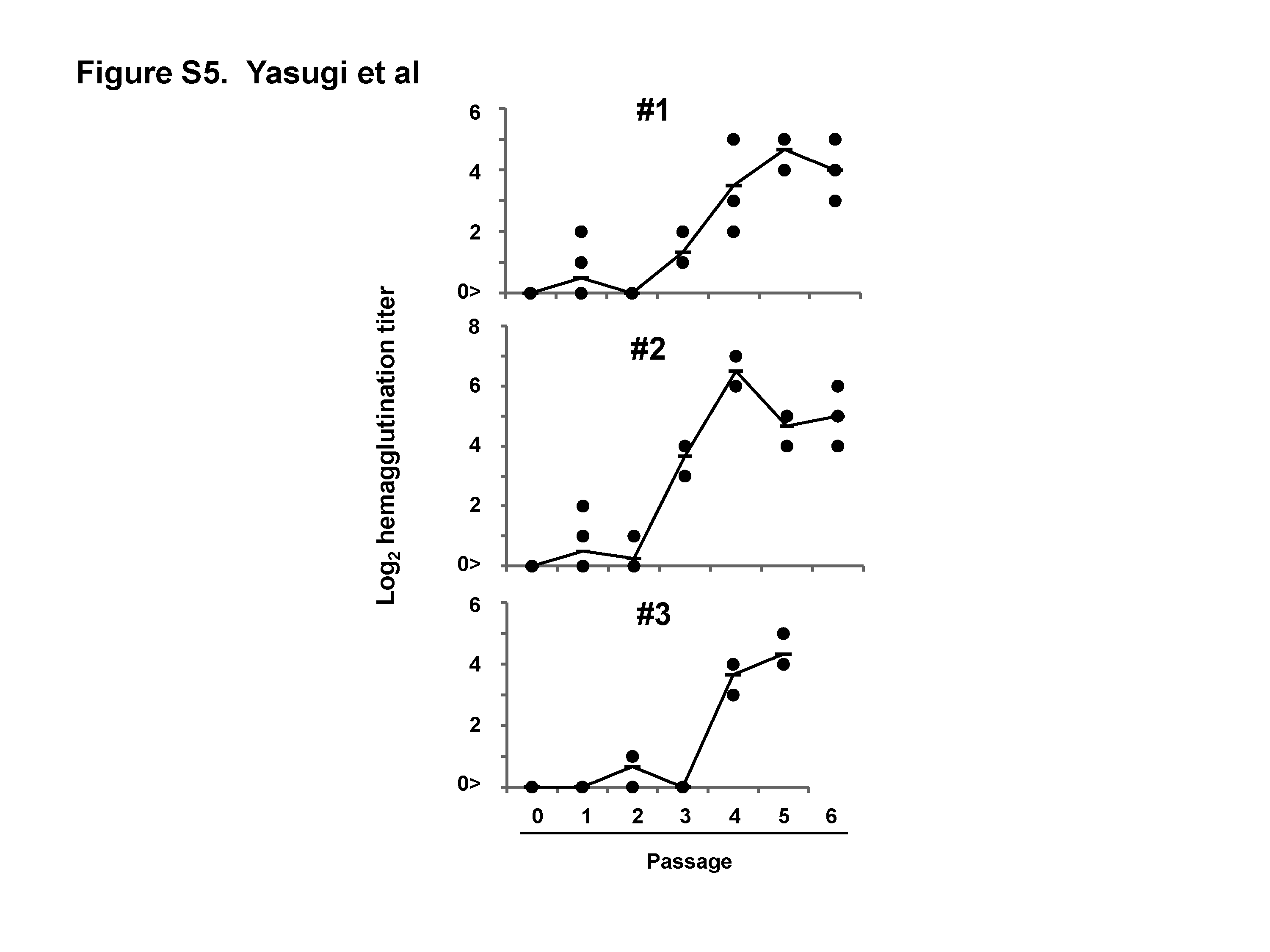

Supplement: Figure S5 — Kinetics of hemagglutination in viruses isolated from egg-passaged samples (#1, #2, and #3). Clinical specimens were injected into 9-day-old embryonated chicken eggs and incubated for 72 h. Allantoic fluid was collected (passage 0) and serially passaged in chicken eggs (passage 5–6). The viral growth of each sample was measured by hemagglutination assay using (0.5%) chicken red blood cells. (TIFF) [file pone.0030946.s005.tiff]

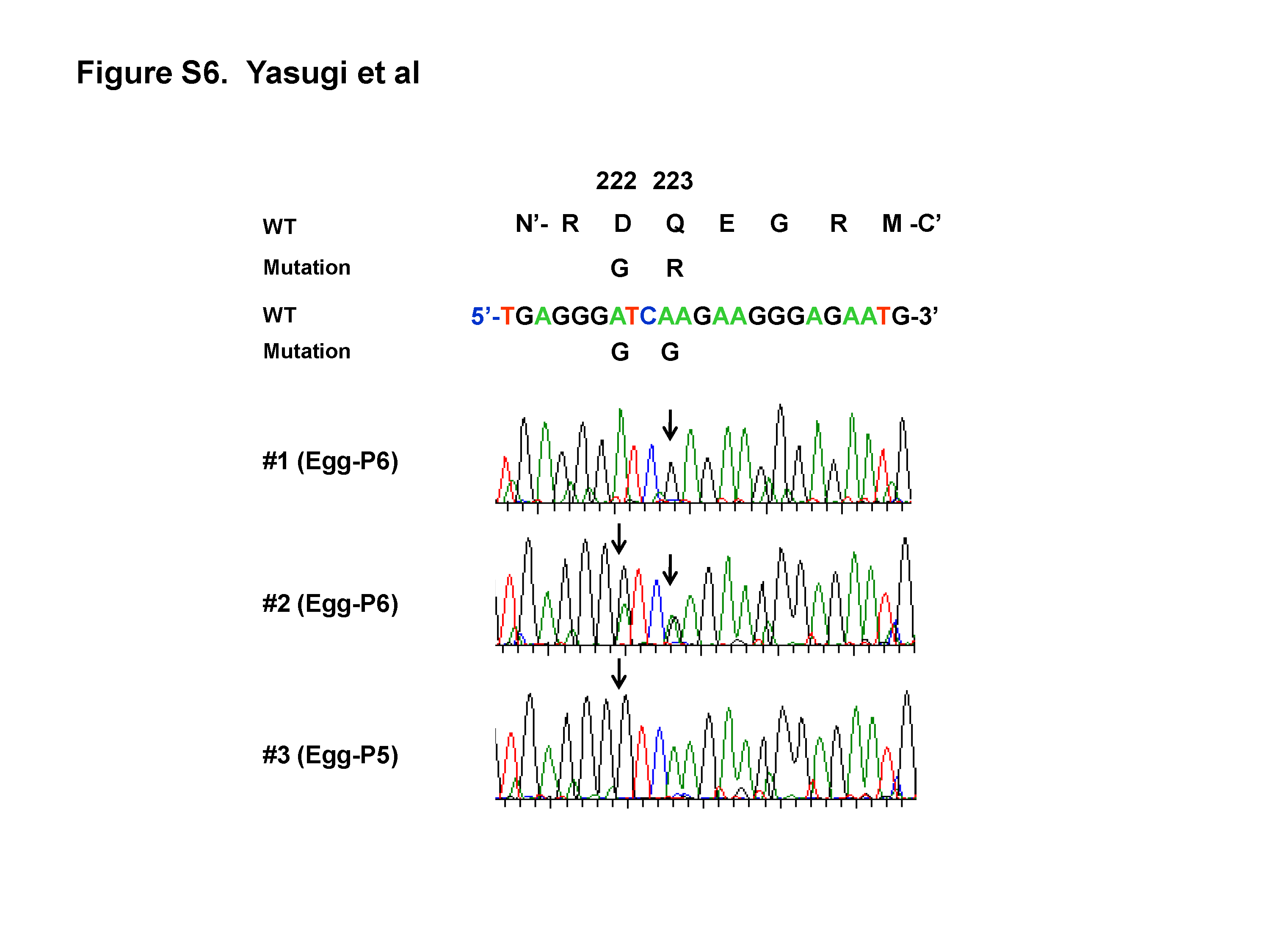

Supplement: Figure S6 — Direct sequencing analysis of PCR products (positions 221–227 aa in HA-RBS) amplified from egg-passaged P6 (#1 and #2) and P5 (#3) viruses using a conventional ABI sequencer. The arrows indicate nucleotide substitutions. WT indicates wild type. (TIFF) [file pone.0030946.s006.tiff]
